# Laryngeal neurofibroma: case report and review of the literature

**DOI:** 10.3389/fonc.2025.1701022

**Published:** 2026-01-27

**Authors:** Yaya Gao, Wei Mo, Hanwen Zhu, Yunhan Zhang, Hui Yang

**Affiliations:** 1 Department of Otolaryngology-Head & Neck Surgery, West China Hospital of Sichuan University, Chengdu, Sichuan, China; 2 West China School of Medicine, Sichuan University, Chengdu, Sichuan, China

**Keywords:** case report and literature review, genetic test, laryngeal neurofibromatosis, microlaryngoscopic excision, NF1

## Abstract

**Background:**

Laryngeal neurofibromatosis (LNF) represents an exceedingly rare manifestation of neurofibromatosis type 1 (NF1), a genetic disorder affecting neural tissue development with an incidence of approximately 1:3000 live births. While NF1 typically presents with cutaneous neurofibromas, laryngeal involvement is exceptional.

**Methods:**

A case of LNF associated with NF1 diagnosed at West China Hospital is presented, accompanied by a comprehensive literature review. A 21-year-old female presented with progressive hoarseness over two years. Diagnostic evaluation included physical examination revealing café-au-lait macules and laryngeal submucosal swelling, and computed tomography (CT) identifying a hypodense parapharyngeal space nodule. The patient underwent microlaryngoscopic excision of the mass. Histopathological examination and genetic analysis were performed.

**Results:**

Histopathology confirmed the mass as a neurofibroma. Genetic analysis identified a pathogenic NF1 gene mutation. Microlaryngoscopic excision was successful, achieving complete removal with minimal intraoperative bleeding and no vocal cord damage, resulting in a favorable postoperative outcome.

**Conclusions:**

This case details the successful diagnosis and management of rare laryngeal neurofibromatosis, confirming microlaryngoscopic excision as an effective approach. The integrated literature review synthesizes current understanding of this rare entity.

## Introduction

Neurofibromatosis, an array of genetic disorders, consists of two distinct autosomal dominant disorders: neurofibromatosis type 1 (NF1) and neurofibromatosis type 2 (NF2) (1). It is typified by irregularities in the development of neural tissue, with an approximate incidence of 1 in 3,000 live births (2). The condition most commonly presents as cutaneous and subcutaneous neurofibromas, benign tumors that affect the skin and underlying tissues (3). Yet, in a minority of cases, these growths may proliferate into deeper anatomical regions, such as the larynx, culminating in a form known as laryngeal neurofibromatosis (LNF) (4).

LNF is exceedingly rare, constituting only 0.03 to 0.1 percent of all benign laryngeal tumors (5). The molecular etiology of LNF is associated with mutations in the NF1 gene, which encodes for a protein pivotal in regulating cell growth (6). Disruptions in this regulatory mechanism can lead to the unchecked proliferation of Schwann cells, resulting in tumorigenesis that may significantly encroach upon and exert pressure on essential laryngeal structures (7, 8). LNF grows very slowly and therefore can present later in life with loss or change of voice, dysphagia, dysphonia, and stridor. This study delineates a case of laryngeal neurofibromatosis enrolled in West China hospital and concurrently furnishes a comprehensive literature review, aspiring to enhance the comprehension of this rare yet consequential disorder.

## Case report

A 21-year-old female was admitted to our hospital with a 2-year history of progressive hoarseness. It is noteworthy that a definitive diagnosis of neurofibromatosis had not been established prior to this presentation. She did not exhibit symptoms such as choking on liquids, difficulty swallowing, a sensation of a foreign body in the throat, or any respiratory distress, including tightness of breath or chest discomfort.

**Figure 1:**
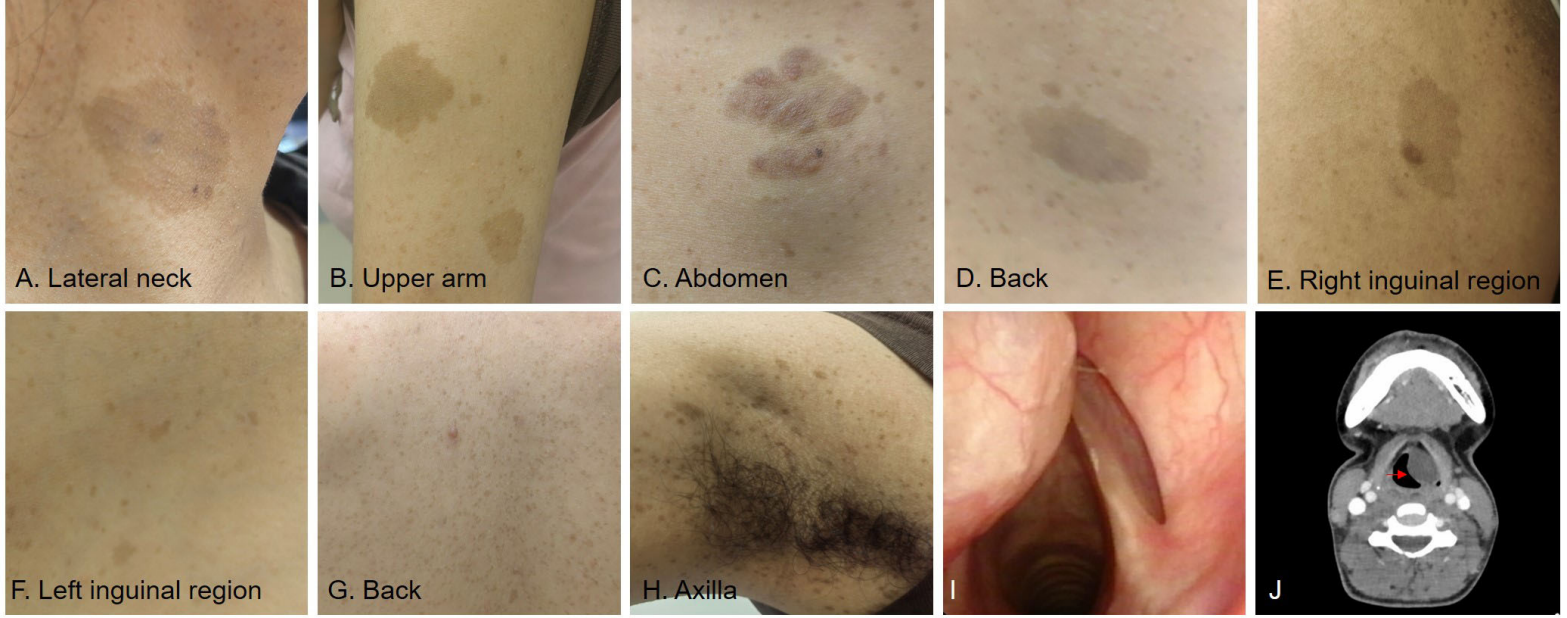
Preoperative physical and specialist examination findings. **(A–E)** Café-au-lait macules were observed on the neck, upper arms, abdomen, back and inguinal region. **(F–H)** Generalized scattered pigmentary macules throughout the body. **(I)**. Laryngoscopy showed a submucosal swelling on the left ventricular band with a smooth surface, obstructing the view of the left vocal cord. **(J)** Computed tomography (CT) scan of the neck showed a slightly hypodense nodule in the left parapharyngeal space, measuring approximately 2.8 x 1.8 cm, with clear boundaries and no significant enhancement.

**Figure 2:**
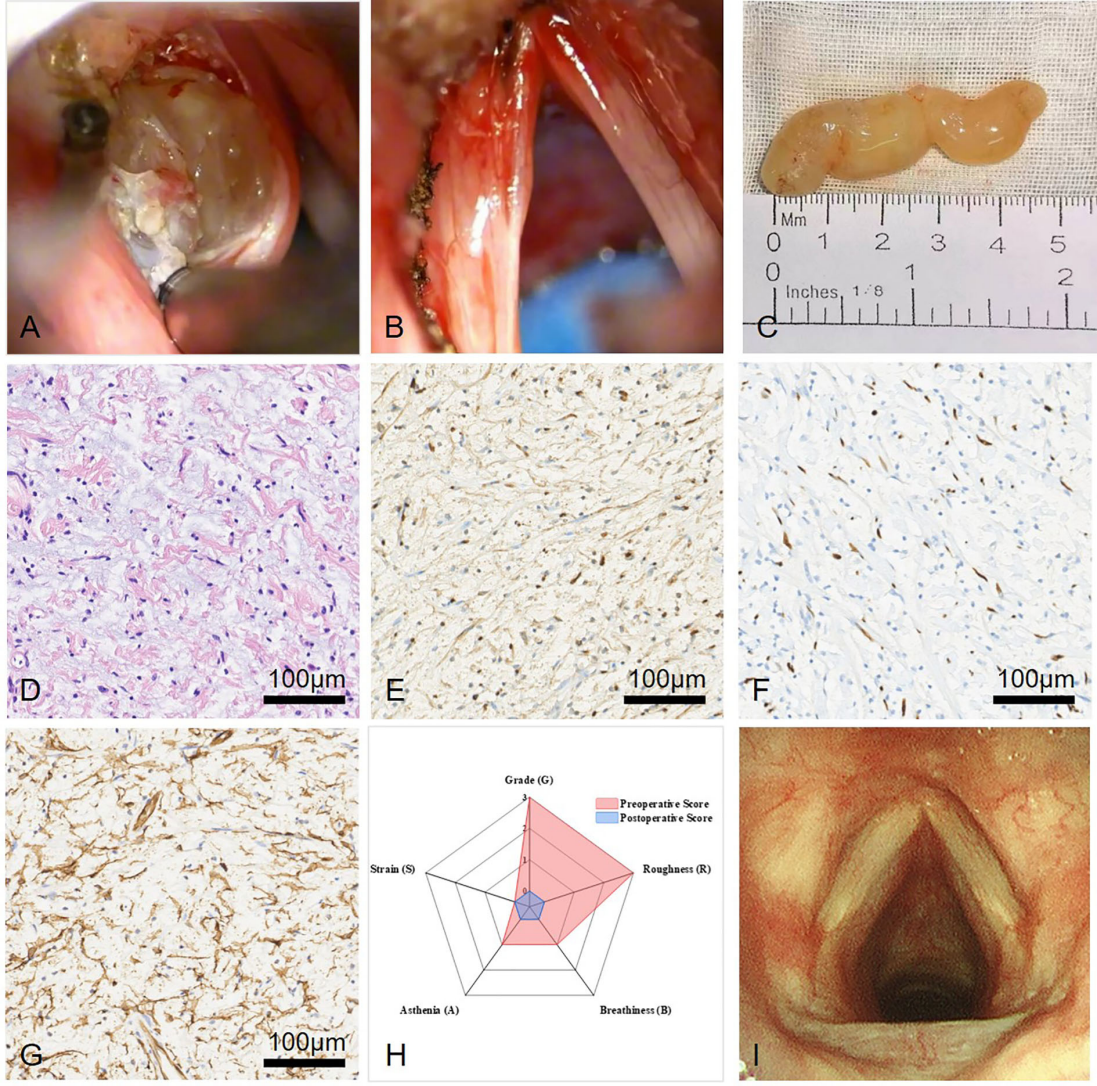
Intraoperative surgery overview, postoperative pathology, and follow-up outcomes. **(A)** Submucosal tumor with clear boundaries and no significant adhesion was separated along the tumor margin. **(B)** Intraoperative findings included a bulging left ventricular band, smooth mucosa of the laryngeal ventricle, and a submucosal yellowish, firm mass measuring approximately 1 cm x 2 cm x 5 cm, encapsulated. **(C)** After complete tumor resection, hemostasis was secured and the wound was cleaned without damaging the vocal cords. **(D)** The majority of the tumor consisted spindle cell proliferation and myxoid degeneration of the stromacytoplasm (HE staining, 20×). **(E)** Immunohistochemistry was positive for S100 (20×). **(F)** Immunohistochemistry was positive for SOX-10 (20×). **(G)** Immunohistochemistry was positive for CD34 (20×). **(H)** Preoperative vs Postoperative GRBAS Scores Comparison. **(I)** No tumor recurrence was found in follow-up laryngoscopy, with both vocal cords having smooth surfaces and normal mobility.

Upon physical examination, she presented with a deep, hoarse voice and difficulty producing high-pitched sounds. Café-au-lait macules (**Figures 1A–E**) were observed on the neck, upper arms, abdomen, and back, accompanied by scattered pigmentary deposits (**Figures 1F–H**) throughout the body. Electronic nasopharyngolaryngoscopy (**Figure 1I**) revealed the left ventricular band was markedly bulging with a smooth surface, obscuring the laryngeal ventricle and vocal cords, partially blocking the glottis. The right ventricular band and vocal cord appeared structurally normal with normal vocal cord mobility, and the glottal chink is slightly narrower than normal. A neck CT scan (**Figure 1J**) showed a slightly hypodense nodule in the left paralaryngeal space, measuring approximately 2.8 x 1.8 cm, with clear boundaries and no significant enhancement. The left aryepiglottic fold, laryngopharynx, and surrounding soft tissues were compressed and displaced, leading to stenosis of the laryngopharynx and the left pyriform sinus. Prior to surgery, a preoperative GRBAS score was completed, with the subjective voice assessment rating as G: 3, R: 3, B: 1, A: 1, S: 0, indicating severe hoarseness (**Figure 2H** red part).

Given that the CT findings indicated a localized tumor with benign characteristics and the comprehensive physical examination suggested a high likelihood of a neurogenic tumor, we opted for transoral endoscopic surgery while also preparing for the possibility of open surgical intervention. Under general anesthesia, a laryngoscope was inserted to fully expose the left ventricular band. Intraoperatively, the left ventricular band was observed to bulge inward, compressing and obscuring the laryngeal ventricle and vocal cords, with a smooth mucosal surface. A plasma incision was made on the mucosal surface, revealing a smooth submucosal tumor that was soft in consistency, well-demarcated from the surrounding tissue, and without significant adhesion (**Figure 2A**). Dissection was carried out along the tumor’s boundary, with extensions of the incision made as necessary, until the medial, lateral, and anterior aspects were completely freed from the surrounding tissue. After the tumor was completely excised, hemostasis was thoroughly achieved and the wound was cleaned, with care taken not to damage the vocal cords or other normal tissues (**Figure 2B**). The surgery lasted 30 minutes, with minimal bleeding of less than 2 ml. The patient recovered well after surgery, with the voice returning to normal on the second day postoperatively, and normal breathing and swallowing functions. There were no significant discomforts such as fever, sore throat, or hemoptysis, and the patient was discharged smoothly on the third day postoperatively.

The tumor measuring approximately 1 cm x 2 cm x 5 cm was completely excised (**Figure 2C**). Postoperative pathological results (**Figures 2D–G**) demonstrated S100 (+), SOX10 (+), H3K27me3 (not lost), CD34 (+), D2-40 (-), p53 (+), p16 (+), SMA(-), Desmin (−), with a Ki67 positive rate of approximately 2%.

One month postoperatively, the patient underwent GRBAS score again, with a subjective voice assessment rating of G: 0, R: 0, B: 0, A: 0, S: 0 (**Figure 2H** blue part), representing a normal voice, marking a significant change from the preoperative condition. At the eight-month follow-up laryngoscopy postoperatively, the laryngeal cavity appeared smooth, with no evidence of recurrence (**Figure 2I**). The patient’s vocal quality was assessed as normal, with no clinically significant dysphonia observed.

**Figure 3:**
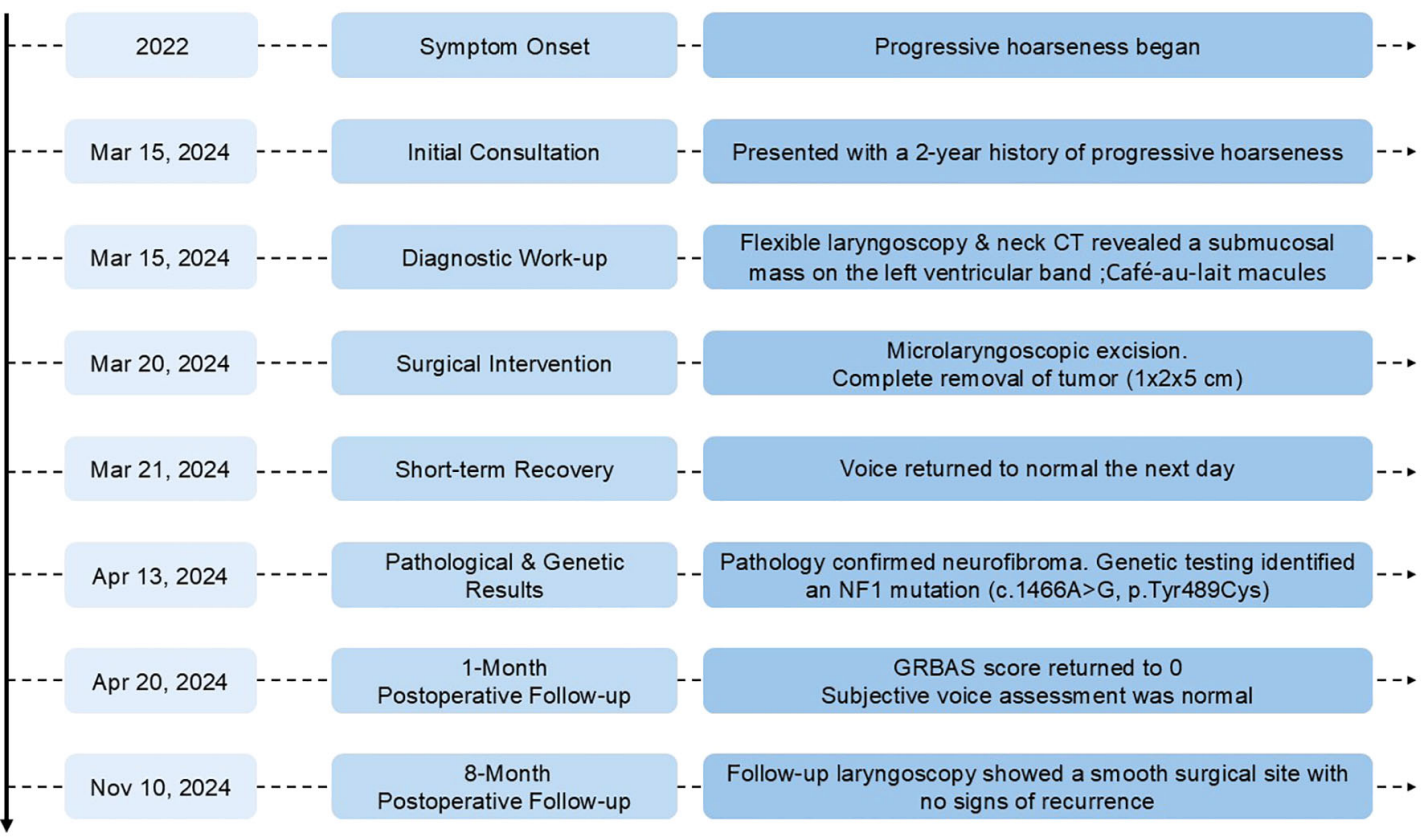
Timeline of diagnosis, treatment, and follow-up for the patient with laryngeal neurofibroma. Key events from symptom onset through postoperative follow-up are chronologically displayed.

To elucidate the diagnosis, genetic analysis was conducted on the patient. The findings disclosed a heterozygous mutation within the NFI locus: a nucleotide transition from adenine to guanine at position 1466 (c.1466A>G), culminating in an amino acid substitution at codon 489, where tyrosine is replaced by cysteine (p.Tyr489Cys). The chronological sequence of key events from initial presentation through surgery and follow-up is summarized in **Figure 3**.

Through a comprehensive assessment of histological diagnosis and genetic testing, the patient was ultimately diagnosed with laryngeal neurofibromatosis.

## Discussion

As previously mentioned, LNF is an exceedingly rare laryngeal tumor that are mainly located in the supraglottic, and awareness of it remains limited. As of July 2024, even though there have been approximately 134 well-documented cases of LNF reported in the literature, timely and effective diagnosis and treatment remain limited. In order to provide a comprehensive elucidation of the most recent epidemiological trends, as well as current diagnostic and therapeutic prospects for LNF, we have delved into the characteristics of laryngeal neurofibromatosis as gleaned from our case series (**Table 1**), highlighting the clinical manifestations, systemic associations, and treatment modalities that define this complex condition (**Table 2**).

**Table 1:** Cases of laryngeal neurofibroma in recent 30 years.

Year	Number of cases	Genders	Age	Location of lesion	Chief complaint	Systemic performance	Treatment modality	Surgical findings	Survival time	Family history	Genetic testing results
1994 (1)	1	Male	8 years old (at the time of tracheostomy)	Supraglottic larynx	Hoarseness	Developmental abnormalities (pectus excavatum, scoliosis)	Surgical excision	Tumor obstructing the laryngeal inlet	***/***	Sibling, mother, grandfather, and great-grandmother affected	*/*
1995 (2)	1	Female	23 years old	Ventricle	Hoarseness	*/*	CO2 laser vestibulectomy, laryngomicrosurgery	Submucosal tumor with a stalk arising from the ventricle	2 years postoperatively asymptomatic	*/*	*/*
1996 (3)	3	Females	*/*	Superior laryngeal nerve	Snoring, episodes of apnea, inspiratory stridor, globus sensation	Multiple café-au-lait spots, Lisch noduli, hamartomas of the iris	Conservative, subtotal resection via lateral pharyngotomy	Tumor involving the left posterior part of the cricoid cartilage, left half of the hyoid bone excised	Two years post-operatively, no breathing or feeding difficulties	*/*	*/*
1996 (4)	1	Male	45 years old	Bilateral ventricles	Harsh and strained voice	Heavy smoker (25 years)	Microlaryngoscopy without tracheostomy	Bilateral polypoid masses	*/*	*/*	*/*
1996 (5)	1 (19th reported pediatric case)	Male	4 years old	Right aryepiglottic fold	Obstructive respiratory symptoms	Poorly developed child, myelomeningocele	Surgical excision	Tumor in the right aryepiglottic fold	*/*	Mother had von Recklinghausen's disease	*/*
1999 (6)	1	Male	39 years old	Supraglottic area	Progressive bilateral hearing loss, dizziness, dyspnea, dysphagia	Bilateral cerebellopontine angle tumors, foremen magnum tumor, spinal cord tumor, retrocervical tumors	KTP laser excision	Submucosal supraglottic tumor	15 months follow-up: no breathing or speech difficulties	*/*	*/*
1999 (7)	7	3 Males, 4 Females	Mean age 6.5 years (range 2 months to 13 years)	Aryepiglottic folds	Various respiratory symptoms	*/*	Partial laryngectomy	Tumor excision including resection of arytenoid and aryepiglottic fold	*/*	7 out of 8 patients had neurofibromatosis	*/*
2001 (8)	1	Male	44 years old	Larynx	Sensation of something in the throat	Normal vocal fold mobility, extensive airway narrowing	Endolaryngeal approach without external incision	Well-circumscribed mass in the right aryepiglottic fold, easily dissected from the mucosa	1 year follow-up with no evidence of tumor recurrence	Mother and other relatives with NF	*/*
2002 (9)	1	Female	35 years old	Larynx and cervical esophagus	Sensation of foreign body in the hypopharynx	*/*	Microsurgery for diagnosis, not all tumors removed	Multiple submucosal tumors	*/*	*/*	*/*
2002 (10)	1	Female	1 year 8 months	Right aryepiglottic fold	Stridor	Cafe-au-lait spots over the trunk and limbs	CO2 laser excision	Solid mass without definite margins during surgery	Follow-up for almost 4 years; asymptomatic	Mother and three sisters with NF-1	*/*
2002 (11)	1	Female	6 years old	Left aryepiglottic fold	***/***	***/***	Lateral pharyngotomy with supraglottic hemilaryngectomy	Solid mass originating from left aryepiglottic fold and prolapsing into the glottic inlet	Followed up for two months with complete cure	*/*	*/*
2004 (12)	1	Female,	4 years old	Left aryepiglottic fold	Progressive inspiratory stridor	No known systemic or congenital abnormalities	Endoscopic surgery using a CO2 laser	2x2-cm pinkish mass on the left aryepiglottic fold obstructing the airway	No recurrence after 4 years of follow-up	No other subcutaneous neurofibromas or café-au-lait spots	*/*
2004 (13)	5	4 girls, 1 boy	At or shortly after birth	Arytenoids and aryepiglottic folds	Stridor and café-au-lait spots at or shortly after birth	*/*	Tracheotomy, carbon dioxide laser excision, modified neck dissection, partial pharyngectomy, supraglottic laryngectomy, endoscopic hemilaryngectomy	Submucosal laryngeal mass, neurofibromas with plexiform and/or diffuse features	*/*	All patients diagnosed with NF1	*/*
2005 (14)	1	Male	35 years old	Subglottic region	Slight exertional dyspnea	Heavy smoker (quit 1 year prior)	CO2 laser resection via microlaryngoscope	Solid mass obstructing tracheal lumen	*/*	*/*	*/*
2008 (15)	2	Males	34 years (case 1), 17 months (case 2)	Arytenoids and aryepiglottic folds	Hoarseness, dyspnea	*/*	Conservative surgery with CO2 laser	Mass partially excised by CO2 laser, irregular cylindrical and fusiform large nervous fascicles	No recurrence after 3 years (case 1), no recurrence after 6 months (case 2)	*/*	*/*
2008 (16)	2	Females	Case 1: 29 years Case 2: 23 years	Larynx and hypopharynx	Change in voice, dysphagia, snoring (Case 1); dyspnea, progressive dysphagia, hoarseness (Case 2)	*/*	Surgical (transcervical resection, lateral pharyngotomy, limited laryngotomy)	Large, exophytic mass obstructing the airway; multifocal and removed in separate components	14-month follow-up: asymptomatic	Case 1: Unremarkable; Case 2: NF-1	*/*
2008 (17)	1	Male	65 years old	Larynx and tracheobronchial tree	Dyspnea and cough	Emphysematous bullae, pulmonary emphysema	Conservative management, respiratory rehabilitation	Multiple neurofibromas in the larynx and tracheobronchial tree	4 months survival post-diagnosis	Positive family history for NF-1	*/*
2013 (18)	1	Female	58 years	Arytenoid	Throat discomfort and dysphagia	*/*	Transoral laser surgery	Hard and smooth mass in the left arytenoid	No recurrence at 18 months	*/*	*/*
2013 (19)	1	Male	78 years old	Vocal fold	Progressive voice hoarseness, intermittent odynophagia	Ischemic heart disease, gastroesophageal reflux	Endoscopic laryngeal microsurgery	Superior cordotomy made lateral to the presumed cyst in the right vocal fold	6 months post-surgery: improved voice	*/*	*/*
2013 (20)	1	Female	56 years old	Arytenoids and parapharynx	Respiratory distress and episodes of apnea at night	Hypertension	Lateral incision approach and transoral carbon dioxide laser	Cervical mass 3.5×5.0cm, yellowish color, ovoid shape, adherent to surrounding tissue	No tumor growth after 2 months, no respiratory or feeding difficulties at 2 years postoperatively	*/*	*/*
2014 (21)	2	1 Male, 1 Female	Case 1: 2 years Case 2: 6 months	Larynx	Persistent sleep apnea, noisy breathing, stridor	Café au lait spots	Surgical (excisional biopsy, tracheostomy)	Cystic mass along the right aryepiglottic fold; solid mass from the level of the vallecula to the false cord on the right	Case 1: Successful decannulation; Case 2: Dependent on tracheostomy tube	Both patients had café au lait spots	*/*
2014 (22)	62 (cumulative from literature)	31 Males, 31 Females	Avg: 4.1 years (range: 0.8-12)	Aryepiglottic fold (most common)	Stridor (most common), dysphagia, dysphonia	*/*	Endoscopic resection, transoral CO2 laser resection	Submucosal mass, often in the supraglottis	*/*	82% associated with NF-1 diagnosis	*/*
2015 (23)	1	Male	5 years old	Neck and Larynx	Exertional inspiratory stridor, throat discomfort	Normal development, no systemic abnormalities	Surgical excision	Large submucosal pinkish mass in the right aryepiglottic fold, bulging into the supraglottic area	24 months follow-up with no recurrence	NF-1 in the patient	*/*
2015 (24)	1	Male	23 years	Aryepiglottic fold	Voice change and throat discomfort for 6 months	*/*	Surgical excision	Well-circumscribed mass without enhancement in the right aryepiglottic fold	*/*	Family history of neurofibromatosis	Confirmed neurofibroma of Type I
2016 (25)	1	Male	11 months old	Post-cricoid region	Hoarseness, weak crying, difficult breathing	Skin "café au lait" spots, hemangioma on the left leg	Endoscopic low-temperature plasma radiofrequency ablation	Submucosal neoplasm in the post-cricoid region	2-year follow-up period with no disease recurrence	Father had multiple milk coffee colored pigmentation spots	*/*
2016 (26)	28	14 Males, 14 Females	Avg: 39.0 years (range: 18.4-63.8)	Various (larynx, vocal cords, etc.)	Vocal weakness (21), dysphagia (5), globus (4)	*/*	Medialization procedures, surgical resection (in some cases)	Various laryngeal pathologies, including visible lesions or weakness	*/*	NF-1 or NF-2 diagnosis preexisting	*/*
2019 (27)	1	Female	30 years old	Left aryepiglottic fold	Severe dyspnea, severe dysphonia, hoarseness, rough voice, diplophonia	Tobacco and betel nut chewing history	Complete laryngectomy	Tumor arising in the left aryepiglottic fold, completely occupying the glottis, damaging the left vocal cord	Postoperatively asymptomatic	***/***	***/***
2019 (28)	1	Male	49 years old	Left vocal cord	Hoarseness of voice, cough	Chronic smoker, non-alcoholic	Microlaryngoscopy with excision	Not mentioned	*/*	No family history of von Recklinghausen disease	*/*
2020 (29)	1	Female	16 years old	Aryepiglottic fold	Snoring, "asthma", dysphagia, OSA	*/*	Microlaryngoscopy and excision with cold steel and CO2 laser	Pedunculated, submucosal lesion at aryepiglottic fold	*/*	*/*	*/*
2020 (30)	1	Female	48 years old	Vocal fold	Progressive dysphonia over 5 years	Reflux disease, obesity	Microlaryngoscopy, cold steel excision	Subcentimeter right vocal fold lesion	*/*	*/*	*/*
2021 (31)	1	Female	22 months old	Larynx	Leukocoria in the right eye, pain, inflammation, vision changes, and exotropia	Cafe-au-lait spots, peripheral non-perfusion in the left eye	Debulking of the laryngeal plexiform neurofibroma	Large left supraglottic, submucosal posterior laryngeal lesion	Stable condition at 6 years old	No family history of NF-1	Pathologic mutation 1246C > T in NF1 gene
2021 (32)	1	Female	54 years old	Right paraglottic space	Progressive dysphonia, dysphagia, globus sensation, increasing dyspnoea	HIV infection, acromegaly, asthma	Laryngoscopy-guided laser resection	Large pedunculated lesion in the right lateral laryngeal wall	*/*	HIV infection, acromegaly, asthma	*/*
2022 (33)	1	Male	3 years old	Right hemilarynx	Stridor, acute airway obstruction	NF1, low-grade cervico-medullary astrocytoma, epilepsy	Coblation (low-temperature plasma radiofrequency ablation)	Large tumor involving right aryepiglottic fold, piriform sinus, ventricle, and false cord	*/*	NF1	*/*
2022 (34)	1	Female	67 years old	Left aryepiglottic fold	Hoarseness and dysphagia, occasional respiratory distress	Kyphoscoliosis, stunted stature, pseudoarthrosis of lower extremities, long-term smoking history	Microlaryngoscopic evaluation, partial excision	Globular mass, soft but not cystic, adherent to mucosa with significant lateral and inferior extent	*/*	NF-1 (Mother also had NF-1)	*/*
2023 (35)	4	3 Males, 1 Female	*/*	Larynx	*/*	*/*	Peroral endoscopic-assisted laryngeal microsurgery	*/*	*/*	Some cases had café-au-lait spots, 1 case with NF-1	*/*
2023 (36)	1	Male	58 years old	Glottis, true vocal fold	Progressive change in voice	Hiatus hernia	Laser-assisted trans-oral microlaryngeal surgery	Right, bulky, smooth surfaced, sub-epithelial bulge along membranous vocal fold	*/*	*/*	*/*
2023 (37)	1	Male	3 months old	Retrocricoid region	Continuous inspiratory stridor, increasing respiratory distress	Prematurity, gastroesophageal reflux	Endoscopic resection	Mobile, soft, rounded mass in the retrocricoid area	12 months follow-up with no evidence of tumor recurrence	Prematurity, gastroesophageal reflux, café-au-lait skin spots	*/*

**Table 2:** Patient characteristics of laryngeal neurofibroma in recent 30 years.

Case	n	134
Age	range	0-78 year-old Mean Age (±SD): 25.6 (±21.3) years
Gender	Male (n, %)	70 (52.24%)
	Female (n, %)	64 (47.76%)
Chief complaint	Respiratory Symptoms (n, %)	61 (45.52%)
	Voice Changes (n, %)	55 (41.04%)
	Pharyngeal Discomfort (n, %)	9 (6.71%)
	Sleep Disorders (n, %)	9 (6.71%)
Systemic performance	Dermatological and developmental abnormalities (n, %)	64 (47.76%)
	Neurological and tumor-related (n, %)	10 (7.46%)
	Not mentioned or other Symptoms (n, %)	60 (44.77%)
Location of lesion	Supraglottic region (n, %)	75 (55.97%)
	Glottic region (n, %)	8 (5.97%)
	Subglottic region (n, %)	2 (1.49%)
	Not mentioned or unclassified (n, %)	49 (36.56%)
Treatment modality	Endoscopic surgeries (n, %)	85 (64.43%)
	Open and excision surgeries (n, %)	35 (26.11%)
	Not mentioned or other surgeries (n, %)	14 (10.44%)
Prognosis	Asymptomatic (n, %)	38 (28.35%)
	Recurrence (n, %)	0 (0.00%)
	Malignant (n, %)	0 (0.00%)
	Not mentioned (n, %)	96 (71.64%)
Family history	Positive (n, %)	76 (56.71%)
	Negative (n, %)	16 (11.94%)
	Not mentioned (n, %)	42 (31.34%)

The epidemiological characteristics of LNF, as derived from our extensive analysis of 134 cases, reveal a disease with a wide age distribution, affecting both pediatric (9) to geriatric (10) populations. This extensive age range is further detailed in the provided table, which indicates that the majority of the cases fall within the 0–78 year-old age bracket, highlighting the disease’s potential to impact individuals across the lifespan. The gender distribution among the cases is fairly balanced, with 70 males (52.24%) and 64 females (47.76%) identified in the study. This balance suggests that LNF does not exhibit a significant gender predilection, which is consistent with the broader understanding of neurofibromatosis type 1 (NF1), a condition that is known to affect both sexes equally (11).

The local manifestations of LNF are diverse and can include respiratory symptoms (12), voice changes (10, 13), pharyngeal discomfort (14, 15), and sleep disorders (16, 17). Firstly, the primary complaints in our series of 134 cases of LNF are predominantly characterized by respiratory symptoms (18–21). These symptoms, which may include shortness of breath, coughing, and chest tightness, underscore the significant impact that LNF can have on an individual’s respiratory function. Secondly, voice changes represent another common presentation (21–23). These changes can range from mild huskiness to complete voice loss and are indicative of the tumors’ potential to affect the function of the vocal cords. The vocal cords are delicate structures responsible for voice production, and any mass effect from a neurofibroma can disrupt their normal movement and coordination. In the specific case mentioned, the patient was admitted to the hospital with hoarseness as the main complaint, which is a direct consequence of the tumor’s impact on vocal cord function. Thirdly, while less frequently reported than respiratory symptoms or voice changes, pharyngeal discomfort and sleep disorders are also part of the clinical picture of LNF (14–17). Pharyngeal discomfort can result from the physical presence of the tumor in the throat, causing a sensation of irritation or a lump. Sleep disorders, such as sleep apnea or insomnia, can arise due to the respiratory disturbances caused by the neurofibroma. Patients with LNF can experience a combination of respiratory symptoms, voice changes, pharyngeal discomfort, and sleep disorders (21–23). The co-occurrence of these symptoms can complicate the clinical presentation and may require a multidisciplinary approach to management.

LNF primarily affects the supraglottic region (16, 24, 25), with a particular predilection for the arytenoepiglottic and/or arytenoid folds due to its rich lymphatic tissue and extensive network of nerves and blood vessels. Under laryngoscopy, LNF typically present as round or oval-shaped tumors with a smooth surface and a solid capsule (4, 21, 26). These tumors are often well-circumscribed and may appear as soft tissue masses within the larynx. Correspondingly, LNF on CT imaging are also characterized by their appearance as well-circumscribed, round, or oval-shaped tumors with a smooth surface and solid capsule (27). They often present as soft tissue masses within the larynx and are typically hypoattenuating and may show minimal enhancement after contrast administration (7). This suggests a composition rich in nerve sheath cells and fibrous tissue (28).

LNF are a distinct manifestation of NF that specifically affect the laryngeal region. Given this, a meticulous and comprehensive physical examination is of significant importance for the diagnosis of LNF. Patients with LNF often exhibit systemic café-au-lait macules, which are among the most characteristic cutaneous features of the condition (15, 27, 29, 30). These macules, along with other clinical features such as neurofibromas, axillary or inguinal freckling, optic pathway gliomas (OPG), more than two Lisch nodules, sphenoid dysplasia, or long bone abnormalities, are part of the diagnostic criteria (31). A thorough evaluation can reveal these features, aiding in the identification of LNF. In particular, the presence of café-au-lait macules and extensive freckling, as seen in the presented case, are key indicators.

The pathological hallmarks of LNF are attributable to its cellular constituent (11). The pathological hallmarks of laryngeal neurofibromas (LNF) are defined by their cellular composition. On hematoxylin and eosin (HE) staining, neurofibromas are characterized by spindle-shaped Schwann cells and fibroblasts interwoven within a collagenous and myxoid stroma, forming a loosely textured neurofibrous matrix. These tumors typically exhibit a low mitotic index, with fewer than 2 mitoses per 10 high-power fields (HPF), and minimal cellular atypia. Immunohistochemically, LNF demonstrate diffuse positivity for S-100 protein, a marker predominantly expressed by Schwann cells in the nervous system. This immunohistochemical feature is indicative of the neural crest origin of the tumor cells (32). Additionally, SOX10, a transcription factor, is also positive in neurofibromas, further supporting their neural crest derivation (11). The identification of a positive fibroblastic network through CD34 immunostaining is characteristic of neurofibromas, highlighting the presence of fibroblasts within the tumor matrix. The tumor does not express cytokeratins, smooth muscle actin (SMA), or desmin (33). Furthermore, neurofibromas typically display a low Ki-67 proliferation index, below 2-5%, which is reflective of their benign nature and suggests a low rate of cellular proliferation (34). The postoperative immunohistochemical profile of this case was highly consistent with the characteristics of LNF.

Family history data reveal a strong positive association in the majority of cases(67.26%), reinforcing the genetic basis of neurofibromatosis and emphasizing the importance of genetic counseling and family screening (20, 25, 35, 36). Genetic counseling is also an important aspect of care for individuals with NF1, as it can help manage the condition and understand the potential for disease progression or complications. In this case, the patient does not have a family history of NF, but genetic testing has identified a mutation in the NF1 gene (26).

Genetic testing plays a pivotal role in the classification of neurofibromatosis. Neurofibromatosis encompasses two distinct types: NF1 and NF2, which share some clinical similarities but have entirely different genetic bases and causative genes (32). NF1 is caused by mutations in the NF1 gene, located on chromosome 17q11.2 (14). This gene encodes neurofibromin, a protein that negatively regulates cell growth and differentiation. Mutations in NF1 lead to uncontrolled cell proliferation, resulting in cutaneous and neurofibromatous lesions. NF2, on the other hand, is caused by mutations in the NF2 gene on chromosome 22q12. The protein encoded by NF2 (37), merlin, plays a crucial role in cell adhesion and growth control. Mutations in NF2 typically lead to bilateral vestibular schwannomas (acoustic neuromas). Genetic testing can precisely identify the mutation types of NF1 and NF2, enabling molecular-level diagnosis and preventing misdiagnoses or missed diagnoses that may occur based solely on clinical presentations.

Treatment modalities are heavily skewed toward endoscopic surgeries (9, 30, 38, 39), reflecting a preference for minimally invasive approaches that may offer reduced morbidity and faster recovery times. Open and excision surgeries remain a viable option for more complex or extensive tumors. 30.5% were resected endoscopically (40), including in this case with the procedure taking only 30 minutes and blood loss being less than 2 ml. The key points of the surgical procedure involve adequate exposure of the tumor, complete resection along its borders, and preservation of healthy tissue. As a novelty in the treatment of this pathology, in 2018, Arnold et al (24) published the first case of LNF treated with robotic surgery transoral in a pediatric patient, with excellent results functional and without recurrence at 5 months of tracking. Because there are few studies carried out and all based on case reports and reviews retrospective, there is no absolute evidence about of the diagnostic algorithm or the treatment gold standard. Prospective studies are limited due to the rarity of the LNF. Complications may occur bleeding, airway obstruction. In some cases it is necessary to perform a tracheostomy, which requires about 40% of patients (19).

A critical consideration in the management of laryngeal neurofibromas is the recognition of histological subtypes, particularly the distinction of the plexiform variant. Unlike their solitary or localized counterparts, plexiform neurofibromas exhibit a diffuse, multi-fascicular growth pattern that infiltrates along the course of nerve branches (32). This subtype is highly characteristic of, and often pathognomonic for, NF1. Of paramount clinical importance is the well-established association between plexiform neurofibromas and a significantly elevated risk of malignant transformation into Malignant Peripheral Nerve Sheath Tumors (MPNST) (32). For individuals with NF1, the lifetime risk of this transformation is estimated to be between 5% and 15% (33). Therefore, identifying a plexiform pattern is not merely an academic exercise but carries direct and serious implications for prognostic stratification, patient counseling, and mandating vigilant, long-term clinical and radiographic surveillance.

The vast majority of patients with LNF experience a favorable prognosis after surgical resection (4, 28, 37, 41). Postoperative follow-up indicates an improvement in clinical symptoms, with no recurrence of the tumor observed (42–45). However, these findings must be interpreted with caution, as they likely reflect limited long-term follow-up data and publication bias rather than an absence of risk. Given the well-documented infiltrative growth pattern of neurofibromas—particularly the plexiform subtype—and the possibility of multifocal disease in NF1, a lifelong potential for local recurrence or new lesion development persists. Symptoms such as hoarseness and dyspnea are significantly ameliorated (40, 46). Long-term follow-up is of utmost importance because of the risk of malignancy (2% to 5%) (47). There is no consensus on frequency, but it should be performed by direct laryngoscopy to warn of recurrences.

## Conclusion

This case is an unusual presentation of NF-1 with laryngeal lesions, and our case series underscored the multifaceted nature of LNF, with a focus on respiratory and vocal manifestations. The predominance of dermatological findings and the supraglottic region involvement reinforces the link to NF1, mandating a multidisciplinary approach to patient care. The preference for endoscopic treatment reflects a trend toward minimally invasive techniques, with the need for continued evaluation of long-term outcomes. The genetic predisposition highlighted by family history data underscores the importance of genetic assessment and counseling in disease management. Future research should aim to provide more detailed phenotypic characterization and long-term outcome data to further refine treatment strategies and prognostic estimations.

## Data Availability

Requests to access the datasets should be directed to YG, gaoyaya_xjtu@163.com.
